# Publisher’s Note

**DOI:** 10.1080/21655979.2024.2326364

**Published:** 2024-03-01

Due to a publisher error, a retraction notice was incorrectly issued on the article ‘The role of second generation sequencing technology and nanomedicine in the monitoring and treatment of lower extremity deep vein thrombosis susceptibility genes’, https://doi.org/10.1080/21655979.2021.2003926. We have now removed the retraction notice.

We apologise to the authors for this error.

